# A prospective survey of *Pseudomonas aeruginosa* colonization and infection in the intensive care unit

**DOI:** 10.1186/s13756-016-0167-7

**Published:** 2017-01-11

**Authors:** Regev Cohen, Frida Babushkin, Shoshana Cohen, Marina Afraimov, Maurice Shapiro, Martina Uda, Efrat Khabra, Amos Adler, Ronen Ben Ami, Svetlana Paikin

**Affiliations:** 1 Head of Infectious diseases unit, Sanz Medical Center, Laniado hospital, Neytanya, Israel; 2 Ruth and Bruce Rappaport Faculty of Medicine, Technion, Haifa, Israel; 3 Infectious diseases unit, Sanz Medical Center, Laniado hospital, Netanya, Israel; 4 Medical and Surgical intensive care unit, Sanz Medical Center, Laniado hospital, Netanya, Israel; 5 National Center of Infection Control, Ministry of Health, Tel Aviv, Israel; 6 Sackler Faculty of Medicine, Tel Aviv University, Tel Aviv, Israel; 7 Infectious diseases unit Tel Aviv Sourasky Medical Center, Tel Aviv, Israel; 8 Microbiology Laboratory, Sanz Medical Center, Laniado hospital, Netanya, Israel

**Keywords:** *Pseudomonas aeruginosa*, Endogenous, Intensive Care unit, Surveillance, ERIC-PCR, Infection control

## Abstract

**Background:**

*Pseudomonas aeruginosa* (PA) surveillance may improve empiric antimicrobial therapy, since colonizing strains frequently cause infections. This colonization may be ‘endogenous’ or ‘exogenous’, and the source determines infection control measures. We prospectively investigated the sources of PA, the clinical impact of PA colonization upon admission and the dynamics of colonization at different body sites throughout the intensive care unit stay.

**Methods:**

Intensive care patients were screened on admission and weekly from the pharynx, endotracheal aspirate, rectum and urine. Molecular typing was performed using Enterobacterial Repetitive Intergenic Consensus Polymerase Chain reaction (ERIC-PCR).

**Results:**

Between November 2014 and January 2015, 34 patients were included. Thirteen (38%) were colonized on admission, and were at a higher risk for PA-related clinical infection (Hazard Ratio = 14.6, *p* = 0.0002). Strains were often patient-specific, site-specific and site-persistent. Sixteen out of 17 (94%) clinical isolates were identical to strains found concurrently or previously on screening cultures from the same patient, and none were unique. Ventilator associated pneumonia-related strains were identical to endotracheal aspirates and pharynx screening (87–75% of cases). No clinical case was found among patients with repeated negative screening.

**Conclusion:**

PA origin in this non-outbreak setting was mainly ‘endogenous’ and PA-strains were generally patient- and site-specific, especially in the gastrointestinal tract. While prediction of ventilator associated pneumonia-related PA-strain by screening was fair, the negative predictive value of screening was very high.

## Background

*Pseudomonas aeruginosa* (PA) is a leading cause of healthcare-associated infections in intensive care units (ICUs), mainly ventilator-associated pneumonia (VAP), central line-associated bloodstream infection (CLABSI) and surgical site infection (SSI). PA colonization typically precedes infection [1]. Colonization may be endogenous, arising from the patient’s own microbial repertoire [2–4], or exogenous if acquired from the hospital environment or by cross-infection from other patients [5–11]. This distinction has implications for the means needed for infection control [12]. Specifically, water fixtures and piping colonized with PA have been implicated as environmental reservoirs during outbreaks in ICUs [13, 14]. Use of point-of-care water filters was shown to effectively reduce PA infections in surgical ICUs [4].

In previous work, we studied the genetic relatedness of PA strains isolated from ventilated patients and hospital faucets. We found a clear temporal and spatial relation between patient and environmental strains [15]. In the present study we aimed to prospectively determine the clinical impact of PA colonization on admission to the ICU and the dynamics of colonization at different body sites throughout the ICU stay.

## Methods

### Study design

The study was conducted at the Sanz Medical Center, a 400-bed community hospital located in central Israel. The adult ICU is a combined medical and surgical unit with ~250 admissions (~2000 patient days) per year. The ICU is located in one room with 6 beds with no physical barrier between patients. ICU staff members were instructed to use tap water for patients bathing only, whereas sterile water was used for drinking, moistening and mouth treatment. All faucet aerators were dismantled 23 months prior to initiation of this study [15].

All patients hospitalized in the ICU from November 2014 to January 2015 were included and underwent prospective weekly PA surveillance cultures, as detailed below. Patients staying in the unit for less than 72 h were excluded from the analysis. The primary endpoint was the development of clinical infection due to PA, defined according to CDC/NHSN surveillance definitions of healthcare-associated infections [16] and American Thoracic Society criteria for VAP [17]. Secondary aims were identifying risk factors for PA colonization on admission and during ICU stay, clonal analysis of strains at each body site during the ICU stay and the concordance between the strains related to infection and those detected on weekly screening.

This study was approved by the hospital institutional review board committee (0033-14-LND). As the study was aimed for infection control and patient safety purposes, the requirement for informed consent was waived.

### Clinical data

The following baseline characteristics were collected from electronic medical records: age, sex, place of residence (home or long-term care facility [LCTF]), comorbidities, hospitalization within 90 days prior to admission, surgery in the previous 30 days and duration of hospitalization before admission to the ICU. We recorded the dates of hospitalization, ICU admission and discharge, Acute Physiology and Chronic Health Evaluation (APACHE) II on ICU admission, length of stay in the ICU and in the hospital in general, ventilation duration, tracheostomy date, death in ICU and within 90 days of hospitalization and major diagnoses in ICU. We also documented the dates and sources of PA cultures (screening and clinical), and PA related diagnoses of VAP, CLABSI, SSI and catheter-associated urinary tract infection (CAUTI).

### Surveillance cultures

Each patient was surveyed using standard bacterial cultures on admission (within the first 72 h) and then once a week until discharge. Cultures were collected using swabs (Transsystem, Copan®, California, USA) from 4 sites: throat, rectum, endotracheal aspirate (EA) for ventilated patients, and urine, and transferred to the laboratory within 30 min. Faucet cultures were collected weekly from the distal part of the faucet using a bacterial swab.

Swabs were inoculated on tryptic soy blood agar, chocolate agar, MacConkey agar and fluid thioglycoate medium (Hy-labs®, Rehovot, Israel). Cultures were incubated at 35° C overnight. Broth samples were subcultured to the same media plates whenever no growth was detected on the initial plates.

Bacterial identification and antimicrobial susceptibility testing were done using the VITEK 2 system (Biomerieux, Marcy l’Etoile, France) and interpreted according to CLSI criteria [18].

PA isolates were stored at -70^o^c for molecular analysis. Molecular typing was done by enterobacterial repetitive intergenic consensus (ERIC)-PCR. DNA was extracted using the easyMag® system (BioMerieux) and ERIC-PCR was performed as previously described [19]. PCR products were resolved using the QIAxcel capillary gel electrophoresis apparatus (QIAGEN, Hilden, Germany) [19]) and compared visually. The discriminatory power of ERIC-PCR was found to be similar to that of PFGE in PA [20].

Acquisition of PA was defined as the isolation of PA from surveillance or clinical cultures from patients not colonized within 72 h of admission. Colonization was defined as the isolation of PA from specimens taken from the rectum, catheter-urine, pharynx or EA, in the absence of clinical infection.

### Statistical analysis

Patient characteristics were presented using descriptive statistics. Continuous variables were compared using the Student t test or Mann Whitney test, and two-tailed Fisher’s exact test was used for categorical variables. Time to PA related infections was evaluated with the Kaplan-Meier method, with the day of ICU admission serving as day 0. Differences between curves were calculated with the two-sided logrank test. Death discharge from hospital, and PA related infection were treated as competing events. In all statistical analyses, a two-sided *p*-value less than 0.05 was considered significant.

## Results

### Faucet samples

Sixty specimens were obtained from 6 faucets over the study period. Of these, only 1 specimen (1.6%) was positive for PA, and was found to be a unique genotype.

### Patient surveillance cultures

**Fig. 1:**
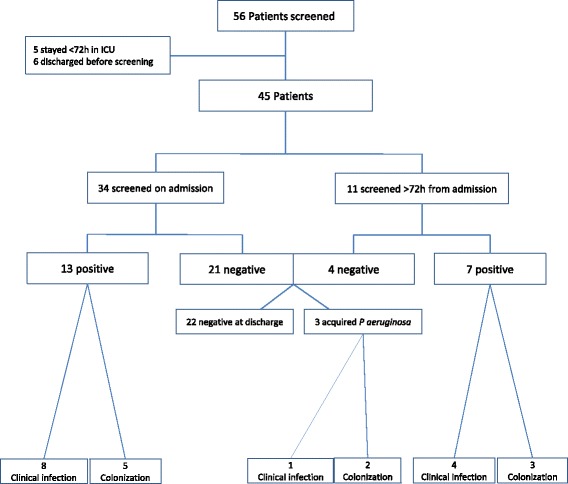
Study population

Fifty-six patients were admitted to the ICU during the study period. Eleven patients were excluded (5 hospitalized < 72h and 6 discharged prior to screening). Out of the remaining 45 patients, 34 patients were screened <72h from admission and 11 were screened ≥72h from admission. Four of the 11 patients screened late were found negative and were regarded also as negative on admission, and together comprised the study cohort of 38 patients (Fig. 1).

Of the 38 patients, 13 (34%) were colonized with PA on admission (Table 1). Advanced age (>70 years) and residency in a LTCF were significantly associated with PA colonization on admission (odds ratio (OR) 7, 95% confidence interval (CI) 1.2-38.3; *p* = 0.035; OR = 17, 95% CI 0.8–358, *p* = 0.033, respectively; Table 1). Diabetes mellitus was negatively associated with PA colonization (OR = 0.06, 95% CI 0.007–0.58; *p* = 0.005).

**Table 1:** Patient characteristics according to *P. aeruginosa* carriage status on ICU admission

	All patients (*n* = 38)	Negative on admission (*n* = 25)	Positive on admission (*n* = 13)	P (OR, 95% CI)
Age mean (range)	70.3 (15-96)	65.6 (15-96)	79.3 (45-94)	0.0039
Age over 70 years	22 (58%)	11 (44%)	11 (84%)	0.035 (7, 1.2-38.3)
Sex				
Male	22 (58)	16 (64)	6 (46)	0.52
Female	16 (42)	9 (36)	7 (54)	0.52
LTCF residency	3 (8)	0	3 (23)	0.033 (17, 0.8-358)
APACHE II score, mean (range)	21.4 (10-42)	21.0 (10-42)	22.1 (14-34)	0.66
Days in hospital until ICU admission, mean (range)	3.7 (0-22)	3.7 (0-22)	3.7 (0-11)	0.98
Hospitalization in the last 90 days	19 (50)	11 (44)	8 (61)	0.49
Surgery in the last 30 days	14 (37)	8 (32)	6 (46)	0.48
Antimicrobials in the last 90 days	17 (45)	12 (48)	5 (38)	0.73
Prior PA in last 90 days	3 (8)	1 (4)	2 (15)	0.26
ICU LOS, mean (range)	15 (3-62)	10.6 (3-39)	23.7 (5-62)	0.0338
Hospital LOS, mean (range)	28 (6-88)	25 (6-80)	33.7 (10-88)	0.14
Ventilation days in ICU, mean (range)	17.3 (0-88)	12.8 (0-79)	26 (0-88)	0.16
COPD	11 (29)	7 (28)	4 (31)	1
IHD	15 (39)	8 (32)	7 (54)	0.29
CHF	14 (37)	7 (28)	7 (54)	0.16
Past CVA	9 (24)	6 (24)	3 (23)	1
CRF	8 (21)	5 (20)	3 (23)	1
Immunosuppression	4 (10)	4 (16)	0	0.27
Dementia	4 (10)	1 (4)	3 (23)	0.1
Active malignancy	4 (10)	3 (12)	1 (7)	1
DM type 2	15 (39)	14 (56)	1 (7)	0.005 (0.06, 0.007-0.58)
Tracheostomy in ICU	12 (31)	6 (24)	6 (46)	0.27
ICU death	5 (13)	2 (8)	3 (23)	0.31
Overall death	13 (34)	8 (32)	5 (38)	0.7

All numbers represent patients (percent), unless specified otherwise. *LTCF* long term care facility, *APACHE*, acute physiology and chronic health evaluation, *ICU* Intensive care unit, *PA P. aeruginosa, LOS* length of stay, *COPD* chronic obstructive pulmonary disease, *IHD* ischemic heart disease, *CHF* congestive heart failure, *CVA* cerebrovascular accident, *CRF* chronic renal failure, *DM* diabetes mellitus, *VAP* ventilator associated pneumonia, *CLABSI* central line associated blood stream infection, *CAUTI* catheter associated urinary tract infection, *OR* odds ratio, *CI* confidence interval

Of the 38 patients in the study cohort, 21 were still hospitalized on the next week, and 11 (52%) of them screened positive for PA (Table 2). The proportion of patients with positive PA screening increased with length of ICU stay, reaching 71% after 3 weeks of ICU stay (Table 2). Three (12%) of 25 patients who were negative on admission screening acquired PA during their ICU stay. In two of them, PA was also found in clinical cultures of sputum, and in one VAP was diagnosed.

**Table 2:** *P. aeruginosa* colonization during ICU stay in 4 screening sites

	Any site (%)	Pharynx (%)	EA (%)	Urine (%)	Rectum (%)
Admission screening (*n* = 38)	13 (34)	3 (23)	6 (46)	1 (7)	10 (77)
Week 1 (*n* = 21)	11 (52)	5 (45)	3 (27)	0 (0)	10 (91)
Week 2 (*n* = 8)	6 (75)	4 (66)	3 (50)	0 (0)	6 (100)
Week 3 (*n* = 7)	5 (71)	4 (80)	4 (80)	1 (20)	4 (80)
Week 4 (*n* = 4)	2 (50)	0 (0)	0 (0)	0 (0)	2 (100)
Week 5 (*n* = 2)	1 (50)	1 (100)	1 (100)	0 (0)	1 (100)
Week 6 (*n* = 1)	0 (0)	0 (0)	0 (0)	0 (0)	0 (0)

*ICU* intensive care unit, *EA* endotracheal aspirate

Of a total of 68 positive surveillance cultures, 33 (49%) were rectal, 17 (25%) pharynx, 16 (23%) EA, and 2 (3%) urine. Rectal screening identified 77% of colonized patients upon admission, 91% after 1 week of ICU stay, and nearly 100% thereafter.

### PA genotyping

During the entire ICU stay we found 20 clonal ERIC-PCR genotypes (among 18 patients) and 11 unique genotypes from 9 patients (two patients had 2 isolates). In the clonal analysis we included cases that were excluded because of being positive on late screening.

**Fig. 2:**
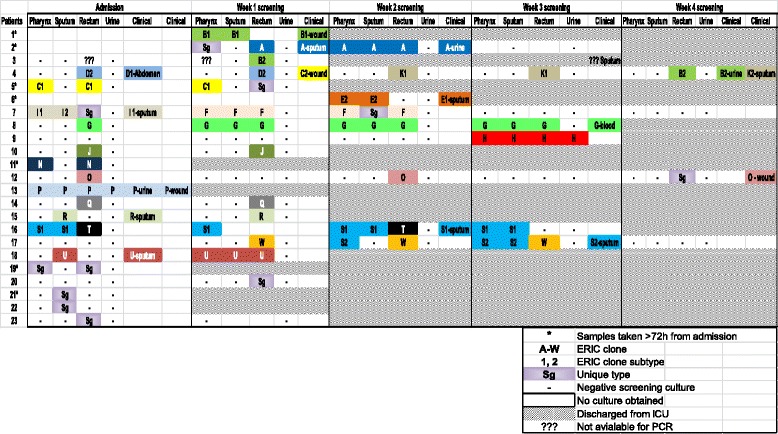
*P. aeruginosa* ERIC-PCR strains among 23 positive patients

Overall, the clonal structure was diverse. There were no dominant strains (related to many patients or to clinical cultures). Twelve patients (patients 2, 4, 5, 7, 8, 10, 12, 14, 15, 16, 17, 18 in Fig. 2) had >1 screening culture (on a following week) available for genotypic analysis (range, 1 to 10 isolates per patient). In 11 of these patients (92%) a serial identical isolate was identified on the following week (all except patient 12, in which the same genotype O was indeed found but only after 2 and 4 weeks, Fig. 2).

### Clonal persistence vs. replacement in sequential screening

Rectum screening: 10 patients had at least 1 sequential rectal screening culture available for typing. In 9 of these (90%) the same clone persisted at least once (range 1–3 weeks). In 3 patients (30%) other strains appeared.

Pharynx screening: 5 patients had at least 1 sequential pharynx screening culture available for typing and in 4 (80%) the same clone persisted at least once (range 1–3 weeks).

AE screening: 4 patients had at least 1 sequential AE screening culture available for typing and in 3 (75%) the same clone persisted at least once (range 1–3 weeks). Cross-overs between sites and strains occurred (Fig. 2).

Calculated together, in 16 out of 19 (84%) of patients in which a sequential same-site screening cultures were available for typing, the same clone persisted. Clonal persistence was evident in all screening sites, but was most prominent in the rectum (90% vs. 80% and 75% in the pharynx and AE, respectively). Cross-overs between sites and strains occurred (Fig. 2).

In 5 patients a spread from the original site of identification to other screening sites was evident (Fig. 2). On 3 occasions the same genotype (B, C, S) was identified in different patients, indicating cross transmission.

### Clinical impact of PA isolation in the ICU

Thirteen patients (29%) were diagnosed with PA infection: 10 with VAP, 4 with SSI and 1 with bloodstream infection. Ten additional patients (22%) acquired PA colonization without infection.

**Fig. 3:**
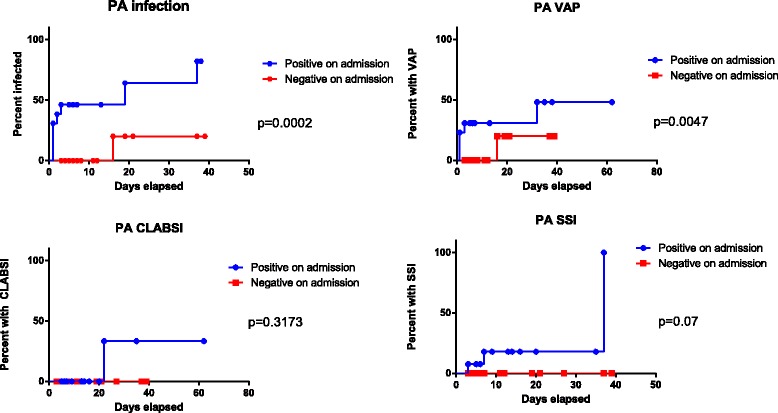
Kaplan-Meier survival curves, comparing PA-related outcomes between positive and negative patients on admission

Patients colonized with PA on admission were at a higher risk of PA-related clinical infection, compared with patients who were PA-negative on admission [8/13 (62%) vs. 1/25 (4%), hazard ratio = 14.65, CI (3.07–47.39), *p* = 0.0002], and for PA-related VAP [hazard ratio = 7.381, CI (1.39–36.41), *p* = 0.0047], Fig. 3). PA-colonized patients also had significantly longer mean stay in the ICU (23.7 days versus 10.6 days; *p* = 0.033, Table 1). None of the 22 patients with repeated negative screening had a positive clinical culture with PA throughout their ICU stay.

Genotyping was performed on 17 clinical isolates from 12 patients (patients 1, 2, 4, 6, 7, 8, 12, 13, 15, 16, 17, 18; Fig. 2). Sixteen (94%) clinical strains were related to strains found concurrently or previously on screening cultures from the same patient and none were unique (Fig. 2, Table 3). The one exceptional clinical isolate (C2 from patient 4) was not unique since it was found in the screening cultures of patient 5). In 6 patients (50%) the clinical PA isolate could have been predicted from the screening cultures between 1 and 4 weeks earlier (genotypes A, K2, G, O, S1, S2); and in the other 6 patients the identification by screening occurred on the same week (genotypes B1, D, I, P, R, U). However, six patients (50%) had screening PA isolates that were different from a concurrent or subsequent clinical PA strain (patients 2, 4, 7, 12, 16, 17). The accuracy of (any site) surveillance cultures to predict the same genotype cultivation in a clinical sample was 76% (36/47), 75% (43/57) and 72% (45/62) when obtained on the same week or within 1 week before, 2 week before and throughout the ICU stay, respectively.

**Table 3:** Concordance between screening and clinical ERIC-PCR strains

	Patient number	Screening culture strain	Site of screening	Clinical culture strain	Clinical culture site	Timing between screen and clinical culture
Correlated cases	1	B1	EA, P	B1	Wound	Same week
	2	A	Rc	A	EA	Same week
			Rc		Urine	1 week
			EA, P, Rc			Same week
	4	D2	Rc	D1	Abdomen	Same week
		K1	Rc	K2	EA	2 weeks
		B2	Rc	B2	Urine	Same week
	6	E2	EA, P	E1	EA	Same week
	7	I1, I2	EA, P	I1	EA	Same week
	8	G	Rc	G	Blood	3 weeks
			EA, P, Rc			2 weeks
			EA, P, Rc			1 week
			EA, P, Rc			Same week
	12	O	Rc	O	Wound	4 weeks
			Rc			2 weeks
	13	P	EA, P, Rc, U	P	Wound	Same week
					Urine	Same week
	15	R	EA	R	EA	Same week
	16	S1	EA, P	S1	EA	2 weeks
			P			1 week
			EA, P			Same week
	17	S2	P	S2	EA	1 week
			EA, P			Same week
	18	U	EA	U	EA	Same week
Uncorrelated cases	2	Sg	P	A	EA	Same week
					Urine	1 week
	4	D2	Rc	C2	Wound	1 week and same week
		B2	Rc	K2	EA	Same week
	7	Sg	Rc	I1	EA	Same week
	12	Sg	Rc	O	Wound	Same week
	16	T	Rc	S1	EA	Same week
	17	W	Rc	S2	EA	Same week

*A*-*W* – *ERIC-PCR* strain (a number denotes a clone subtype), *Sg* unique strain, *EA* endotracheal aspirate, *P* pharynx, *Rc* – rectum, *U* urine

Table 4 shows the correlation of the site-specific screening culture with the infective strain according to the different diagnoses. Among 8 patients with VAP (who had clinical AE cultures available for typing), identical surveillance cultures were recovered from EA in 7 (87%), from the pharynx in 6 (75%), and from the rectum in 4 (50%). Among 4 patients with SSI, identical surveillance cultures were recovered from EA, pharynx and rectum in 2 patients (50%) each.

**Table 4:** Prediction of clinical strain by screening sites according to diagnosis

Diagnosis	Clinical clone	Concordant screening sites				Discordant screening sites			
		EA	P	Rc	U	EA	P	Rc	U
VAP	A	+	+	+			+		
	K2			+				+	
	E1	+	+						
	I1	+	+					+	
	R	+		+					
	S1	+	+					+	
	S2	+	+					+	
	U	+	+	+					
Screen site utility for VAP (%)		7/8 (87)	6/8 (75)	4/8 (50)	NA	NA	1/8 (12)	4/8 (50)	NA
SSI	B1	+	+						
	C2							+	
	O			+				+	
	P	+	+	+	+				
Screen site utility for SSI (%)		2/4 (50)	2/4 (50)	2/4 (50)	1/4 (25)	NA	NA	2/4 (50)	NA
BSI	G	+	+	+					
IAI	D1			+					
Screen site utility for all infections (%)		10/14 (71)	9/14 (64)	8/14 (57)	1/14 (7)	NA	1/14 (7)	6/14 (43)	NA

*VAP* ventilator associated pneumonia, *SSI* surgical site infection, *BSI* blood stream infection, *IAI* intraabdominal infection, *EA* endotracheal aspirate, *P* pharynx, *Rc* rectum, *U* urine

## Discussion

We used systematic sequential screening to define the dynamics of PA colonization and infection at a general ICU. In a non-outbreak setting, we found a highly diverse population of patient-unique PA strains. Strains were often site-specific and site-persistent, particularly with regards to rectal colonization, but could also distribute between body sites, and be replaced frequently. A positive screening culture for PA was associated with an increased risk of PA related infection: there was a 50–70% likelihood of subsequent clinical infection with the same strain, depending on the timing and site of screening. Importantly, we found that when adequate infection control standards are maintained, repeated negative multi-site screening results were associated with a very low rate of subsequent clinical infection with PA.

A third of our patients were carriers of PA on admission to the ICU (26% rectal, 16% EA and 8% pharyngeal carriage). Bonten et al. reported similar figures (34%) along with striking similarities regarding the relative importance of the sites of screening: the gastrointestinal being the most sensitive (24% positivity), and pharynx and EA being positive in only 9% and 7%, respectively [3]. In a more recent study, Zorilla et al. reported similar findings (27% PA colonization on admission) [21]. Advanced age and prior hospital stay were risk factors for PA colonization on admission. Similarly, we found advanced age and residence in a LTCF as significant risk factors. Surprisingly, diabetes mellitus was associated with a low rate of PA colonization on ICU admission. In line with others [1, 22], we found that colonization often preceded infection. Specifically, patients colonized upon admission had a 14.65-fold risk of developing infection as compared with non-colonized patients.

Early and accurate antibiotic coverage in patients developing VAP in the ICU is critical to improve patient outcomes [23, 24], but the increasing rates of multidrug resistant (MDR) organisms (including PA) in ICU and non-ICU patients pose an obstacle for appropriate empiric therapy. Accurate prediction of antimicrobial resistance patterns of organisms causing VAP by using surveillance cultures in ICUs has been a matter of an ongoing debate in the literature. A recent systematic review and a meta-analysis found high accuracy of surveillance cultures, with pooled sensitivities of up to 0.75 and specificities up to 0.92 in culture-positive VAP [25]. Our results support the predictive value of surveillance cultures: among patients who developed VAP, screening the EA or the pharynx accurately predicted the VAP-related strain in 75–87% of episodes. SSI-related strains were predicted by EA and pharynx screening in 50% of cases.

None of the patients who had persistently negative surveillance cultures had subsequent recovery of PA from clinical cultures. Similar findings were reported in the meta-analysis cited [25]. Hence, screening two sites weekly with negative results can provide reassurance for the physician not to initiate empirical anti-pseudomonal antibiotics in patients with suspected VAP or SSI, which are among the most frequent infections in critically ill patients. This finding may have implications for antibiotic stewardship, as it provides an evidence-based framework for limiting the use of wide-spectrum antibiotics in the ICU.

The current study is unique in providing a longitudinal assessment of PA colonization dynamics in multiple body sites throughout the ICU stay. Recently, Zorrilla et al. [1] found high rates (87%) of genotypic concordance between rectal surveillance cultures and infecting strains of PA. Our results underscore the limitations of rectal screening for predicting respiratory strains, as further demonstrated in a study performed among hematopoietic stem cell recipients [26]. The high efficacy of lower airways screening to predict the strains that caused VAP is consistent with results of previous studies [3, 10].

The limitations of this study are the relatively small number of patients in a single center setting. Screening was limited to PA colonization, whereas in clinical practice empiric antimicrobial therapy often targets other MDR bacteria such as MRSA, MDR-*Acinetobacter* spp. and ESBL-producing Enterobacteriaceae. From a practical perspective, screening 3 body sites for PA only, may be expensive and labor intensive, and will miss other important causes of VAP and SSI. Another limitation is that antimicrobial susceptibility data of all screening strains was not available for comparison. Therefore, the utility of screening cultures to predict the susceptibility patterns of clinical PA strains remains to be established.

## Conclusions

In this study we showed that in a non-outbreak setting of ICU, most strains were patient-unique, endogenous in origin, and cross contamination was rare. Colonization on admission was a significant risk factor for the development of infection with PA. Detection of PA on surveillance cultures may serve as a good predictor of PA clinical infection and also of the infecting clone, while negative screening is an excellent negative predictor for clinical infection. VAP-related strains are better predicted by upper airways screening than rectal screening.

## Abbreviations

APACHE: Acute physiology and chronic health evaluation
CAUTI: Catheter-associated urinary tract infection
CDC: Centers for disease control and prevention
CLABSI: Central line-associated blood stream infection
EA: Endotracheal aspirate
ERIC: Enterobacterial repetitive Intergenic Consensus
ICU: Intensive care unit
LCTF: Long-term care facility
MDR: Multidrug resistant
NHSN: The national healthcare safety network
PA: *Pseudomonas aeruginosa*
PCR: Polymerase chain reaction
SSI: Surgical site infection
VAP: Ventilator associated pneumonia
